# Case report: Concurrent low-volume subdural hematoma and ipsilateral ischemic stroke presenting as capsular warning syndrome: a complex case with anticoagulation dilemma and dual pathology

**DOI:** 10.3389/fneur.2024.1358237

**Published:** 2024-02-20

**Authors:** Daniel Strahnen, Angeliki Stathi, Jürgen Beck, Roland Roelz, Ioannis Vasilikos

**Affiliations:** Department of Neurosurgery, University Medical Center Freiburg, Freiburg, Germany

**Keywords:** capsular warning syndrome, subdural hematoma (SDH), ischemic stroke, antithrombotic treatment, computer tomography (CT), magnetic resonance imaging (MRI)

## Abstract

**Background:**

The simultaneous emergence of low-volume subdural hematoma and ipsilateral ischemic stroke in an atrial fibrillation patient who is under anticoagulation therapy is a rare and intricate clinical case. This report accentuates the diagnostic and treatment complexities associated with these consecutive neurological conditions.

**Case presentation:**

An 83 years-old male patient initially presented with acute dyspnea, raising the suspicion of pulmonary embolism. After exclusion of pulmonary embolism through CT angiography, the patient experienced a sudden onset of left-sided hemiparesis without prior history of head trauma but with chronic intake of apixaban due to atrial fibrillation. Subsequent cranial CT tomography revealed a small right parietal subdural hematoma. After reversal of the anticoagulation therapy, surgical evacuation of the subdural hematoma was successfully performed. However, in the postoperative period, the patient developed new neurological symptoms that could not be explained by the reduced size of the subdural hematoma on a follow-up CT scan. Cranial MRI revealed the coexistence of acute ischemic stroke in the right corona radiata. The recent surgical procedure precluded guideline-recommended stroke treatment.

**Discussion:**

This case underscores the complexities of diagnosing and treating concomitant small volume subdural hematoma and ischemic stroke, especially if the latter occurs in the corona radiata resulting in fluctuating symptoms known as “capsular warning syndrome.” Reversal and secondary discontinuation of anticoagulant therapy for surgical intervention highlight the inherent risk of thrombotic events in anticoagulated patients. The development of tailored treatment strategies requires a multidisciplinary approach, and further research and guidelines are required in similar complex scenarios.

**Conclusion:**

The presence of both a small subdural hematoma and an ipsilateral ischemic stroke presenting as capsular warning syndrome in an anticoagulated patient highlights the intricacy of their care. This case calls for a comprehensive and collaborative strategy to address complicated clinical scenarios.

## Introduction

Subdural hematoma and ischemic stroke are separate neurological emergencies, each with distinct diagnostic criteria and therapeutic considerations. The simultaneous occurrence of both conditions in a single patient, although infrequent, poses a significant clinical complexity. The potential risk for subsequent stroke is introduced as antithrombotic medications are often discontinued when surgical evacuation of the subdural hematoma is indicated.

Subdural hematomas stem from blood accumulation between the dura mater and arachnoid membrane, which vary in acuity from acute to chronic (1). Diagnosis of subdural hematomas relies on comprehensive neurological assessment and neuroimaging studies, including computed tomography (CT) or magnetic resonance imaging (MRI), to determine the size, location, and chronicity (2). Hematoma management is dictated by the characteristics of the hematoma and its clinical presentation. Smaller asymptomatic hematomas may be monitored conservatively, while larger or symptomatic hematomas often require surgical evacuation. Different methods for surgically evacuating subdural hematomas are available; however, there is no conclusive evidence indicating the superiority of a specific approach (3). Unfortunately, surgical intervention frequently mandates the discontinuation of antithrombotic medications, which poses a clinical dilemma and may increase the risk of subsequent thrombotic events (4).

Rapid and accurate diagnosis is imperative for ischemic stroke, which arises from the occlusion of the cerebral arteries. Diagnostic procedures involve clinical assessment and imaging tests like CT or MRI, as well as advanced methods such as CT angiography or magnetic resonance angiography to identify vascular occlusions or stenoses (5). The management of ischemic stroke has significantly progressed with the essential administration of intravenous thrombolysis and endovascular thrombectomy when appropriate. The goal of these interventions is to re-establish blood flow to the affected brain tissue, but they may entail specific risks and require a cautious approach, particularly in patients with prior utilization of antithrombotic medication (6). Capsular warning syndrome (CWS) is a unique entity characterized by recurrent transient ischemic attacks (TIAs) that typically present with motor or sensory symptoms, heralding a significant risk of imminent stroke, often within the initial days following the first TIA episode. Considering the critical nature of CWS, it is essential to underscore the urgency for prompt identification and rapid intervention in cases of recurrent TIAs. Timely antithrombotic treatment plays a pivotal role in clinical practice, given the substantial risk of recurrent stroke in the short term following a TIA episode, as an immediate and robust response was shown to be necessary to prevent the progression to irreversible cerebrovascular events by swift diagnostic and therapeutic measures (7, 8). Despite the urgency associated with CWS, there remains a notable gap in the literature regarding the definitive effectiveness of various treatment strategies, such as single antiplatelet therapy (SAPT), dual antiplatelet therapy (DAPT), intravenous thrombolysis (IVT), and anticoagulants, in preventing progression to irreversible stroke. This uncertainty stems from the fact that current management guidelines are largely based on observational studies rather than randomized controlled trials (RCTs), rendering treatment approaches speculative to a certain extent (7, 8). Therefore, our case report not only contributes to the body of knowledge on the rare co-occurrence of CWS and SDH and its complex clinical management but also serves as a critical reminder of the urgency required in the clinical management of TIAs. By highlighting these aspects, especially considering the need to halt antithrombotic medications during surgical evacuation of the hematoma, we aimed to reinforce the importance of early recognition and intervention in such cases, which could be pivotal in mitigating the risk of adverse outcomes in patients presenting with recurrent TIAs.

This study examines the diagnostic complexity and treatment choices encountered by healthcare providers when handling this distinct clinical situation.

## Case description

An 83 years-old male patient with a medical history of atrial fibrillation was undergoing apixaban anticoagulation therapy. The only known preexisting conditions were alcohol abuse and chronic folic acid deficiency. He was admitted to an external hospital for acute dyspnea and was diagnosed with suspected pulmonary embolism. However, computed tomography (CT) angiography of the thorax negated the diagnosis of pulmonary embolism as the cause of the patient’s dyspnea. The plasma clotting was normal during the initial assessment.

**Figure 1:**
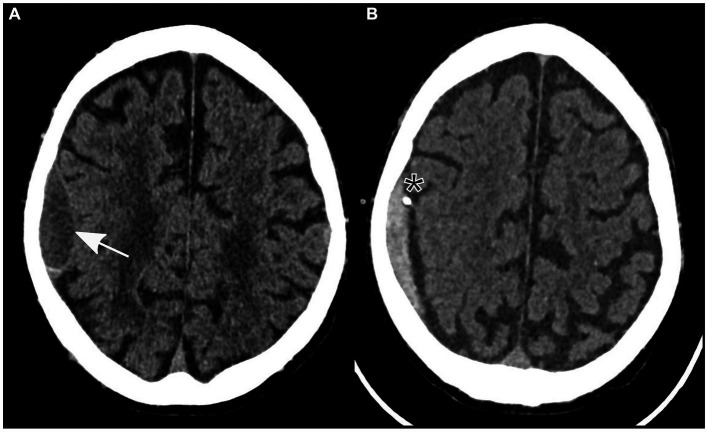
Non-contrast axial computed tomography showing a subdural hematoma in the parietal lobe (identified by an arrow) prior to **(A)** and following insertion of a subdural drain (asterisk) through a twist-drill craniostomy **(B)**.

Further examination was necessary after the patient experienced sudden left-sided hemiparesis that fluctuated in severity following elimination of a pulmonary embolism. There was no sign of a traumatic event at any point during clinical presentation. A non-contrast cranial CT scan was conducted, which indicated a low-volume subdural hematoma localized to the right parietal hemisphere (Figure 1A). Importantly, no evidence of other intracranial pathologies was detected in the initial CT scan. In addition, the patient reported no relevant headaches, nausea, or vomiting.

Owing to the severity of the patient’s neurological findings, he was transferred to our tertiary referral hospital for specialized care. A twist-drill craniostomy was performed, and a subdural drain was inserted parietally in the right hemisphere due to the size of the subdural hematoma and its neurological symptoms. In preparation for the surgical procedure, 3,500 international units of prothrombin complex concentrate were administered to safely reverse the effects of anticoagulation therapy.

**Figure 2:**
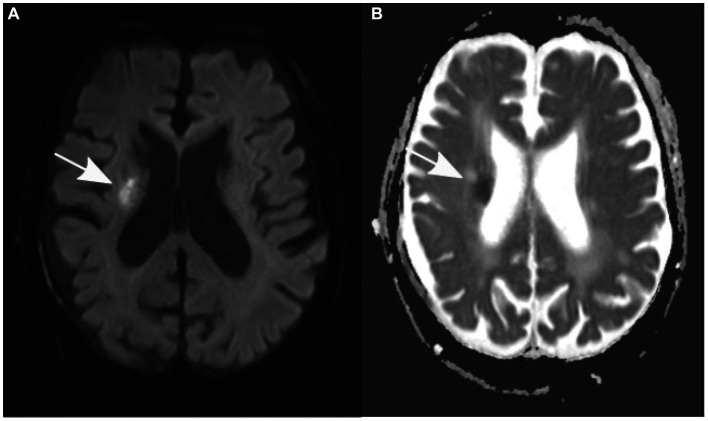
Axial diffusion-weighted magnetic resonance images **(A)** show an elevation in signal, while apparent diffusion coefficient values **(B)** are decreased in the right corona radiata (indicated by an arrow).

After the patient underwent successful surgery, he was transferred to the standard ward for postoperative monitoring. Nevertheless, the following morning, the patient displayed new neurological symptoms, such as dysarthria and exacerbated left-sided hemiparesis. A subsequent cranial CT scan verified the correct positioning of the subdural drain and revealed a decrease in the size of the subdural hematoma (Figure 1B). Sudden onset of dysarthria and worsening hemiparesis on the left side during the postoperative phase necessitated further imaging. Follow-up cranial CT revealed a decrease in the size of the subdural hematoma; however, this could not explain the aggravated neurological symptoms. An MRI scan of the brain revealed the coexistence of an ischemic stroke in the hyperacute phase of the right corona radiata in the supply area of the right anterior choroidal artery. It exhibited increased brightness on diffusion-weighted imaging (DWI) and reduced apparent diffusion coefficient (ADC) values (Figure 2). Further neurological assessment revealed moderate stenosis of the left middle cerebral artery in the distal M1 segment. A pivotal clinical challenge that has emerged is the lack of clear guidelines on the timing of antithrombotic restart following the occurrence of SDH. This challenge was further compounded by the high risk of very early stroke (within 24 h) in our patient, who presented with recurrent TIAs suggestive of CWS. Given this high risk, the immediate initiation of secondary prevention is a critical consideration. However, considering the recent surgery and the absence of large intracranial artery occlusion, no antithrombotic treatment was initiated.

**Figure 3:**
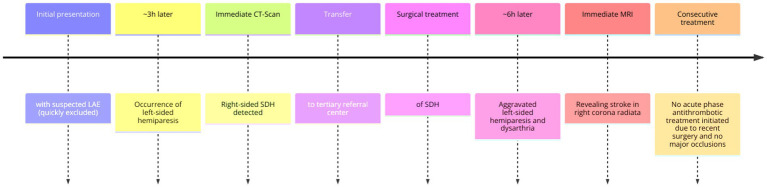
Approximate timeline of events. LAE, lung artery emboly; CT, computer tomography; SDH, subdural hematoma; MRI, magnetic resonance imaging.

This limitation highlights the challenges posed by the sequential occurrence of these two neurological conditions and their associated therapeutic decisions (see Figure 3).

## Discussion

The patient’s history of atrial fibrillation and apixaban use was a crucial factor in this case. The reversal of anticoagulation treatment to prepare for subdural hematoma evacuation emphasizes the inherent risk of thrombotic events in these patients. While atrial fibrillation mandates anticoagulation for stroke prevention, such anticoagulation also presents potential obstacles during surgical procedures.

It is important to note that while the patient initially presented with symptoms indicative of lacunar syndrome, specifically pure motor hemiparesis, subsequent investigations revealed that these symptoms were not the result of a lacunar infarct. This distinction is critical, as lacunar syndromes are commonly associated with small deep cerebral infarctions; however, as demonstrated in our case and supported by the existing literature, they do not always signify lacunar infarcts. This phenomenon, where lacunar syndromes are not attributed to lacunar infarcts, has been documented in the medical literature, with studies indicating that such cases account for approximately 16.6% of lacunar syndrome presentations (9). In the context of our case, understanding this distinction is pivotal for guiding our diagnostic and therapeutic approaches. The initial presentation of pure motor hemiparesis could have led to a presumptive diagnosis of a lacunar infarct; however, further investigations pointed towards a different pathology, underscoring the importance of thorough clinical and radiological assessment in cases with such presentations. This case highlights the need for clinicians to be aware of the diversity in the presentation of lacunar syndromes and the potential for non-lacunar pathologies to present with similar symptoms. This reinforces the importance of comprehensive diagnostic processes in stroke-like presentations, particularly in complex cases with coexisting pathologies, as observed in our patient.

Furthermore, the symptoms of our patients were characterized by episodic sudden onset weakness with partial resolution of symptoms in between. His symptoms and signs were becoming persistent despite the addition of surgical evacuation of the subdural hematoma. The history we describe is classical of capsular warning syndrome (CWS). Capsular warning syndrome is a rare clinical syndrome characterized by recurrent and frequent transient episodes of focal neurological deficits with a high risk of infarction (10). The exact physiological mechanism of CWS remains unclear, but it is most commonly believed to be a result of hemodynamic insufficiency in diseased small penetrating vessels (11). As many as 71% of patients with CWS eventually develop permanent infarction, little has been published about its prognosis, management strategies, and treatment outcomes. Studies have shown that the mean duration of recurrent TIA episodes in CWS varies from 6 to 24 min, and the internal capsule remains the most frequently involved location of established infarct on MRI in 50%–70% of cases (12).

In contemplating the intricate interplay between intracranial pressure (ICP) dynamics and stroke aetiology, one intriguing hypothesis arises: could hemodynamic alterations driven by elevated ICP contribute to the occlusion of small penetrating arteries, thereby leading to lacunar stroke? Theoretically, increased ICP may affect cerebral perfusion, particularly in small vessels, which are critically dependent on stable perfusion pressures. Such hemodynamic instability could potentially result in reduced blood flow or even trigger vasoconstriction, potentially predisposing patients to lacunar infarcts. However, it is important to recognize that current scientific literature does not provide substantial direct evidence linking increased ICP specifically to lacunar stroke via these mechanisms. This gap highlights the necessity for more targeted research exploring how fluctuations in ICP might influence the pathophysiology of lacunar stroke. Investigating this relationship could yield pivotal insights into stroke mechanisms, and foster the development of nuanced prevention and treatment strategies. Our case adds to this discussion by presenting a scenario where such hemodynamic considerations might be relevant, underscoring the need for further exploration of this complex and potentially significant aspect of stroke pathophysiology.

Regarding the possible limitations of the present case, as with any case report, the findings presented here are based on the unique clinical presentation and management of a single patient, which may not be broadly applicable to other patients with similar conditions. The rarity and complexity of the patient’s presentation, involving simultaneous subdural hematoma and ischemic stroke during anticoagulation therapy, may limit the applicability of our findings to more common clinical scenarios. The diagnostic and treatment approaches employed in this case were constrained by available resources and may not reflect the full spectrum of options available in different clinical settings. For example, it could be argued that the worsening of hemiparesis was just another fluctuation in the context of CWS; therefore, a FLAIR negative MRI indicating a <4 h stroke might have been convincing evidence for separate events, whereas the CT scan, as in our case, was not sensitive enough in the acute phase in such locations. In addition, the potential influence of unreported or unidentified confounding variables, such as genetic predispositions or other comorbidities, cannot be ruled out in this case.

As this case report sheds light on the complexities involved in managing concurrent low-volume subdural hematoma and ipsilateral ischemic stroke in a patient undergoing anticoagulation therapy, it opens several avenues for future research. First, large-scale studies are needed to better understand the incidence and outcomes of such dual pathologies, which will aid in the development of more refined management protocols. Additionally, the role of anticoagulation in patients with coexisting conditions that warrant and contraindicate its use presents a significant clinical challenge. Research focused on the optimization of anticoagulation strategies in such scenarios is crucial. Furthermore, the potential genetic, physiological, and pharmacological factors that contribute to the development of such concurrent pathologies warrant further investigation. Lastly, long-term follow-up studies are essential to assess the outcomes and quality of life of patients treated for simultaneous occurrence of stroke and subdural hematoma, particularly in the context of anticoagulation therapy. By addressing these research gaps, we hope to enhance clinical decision making and improve patient outcomes in similarly complex cases.

## Conclusion

This case report highlights the challenges in managing patients with both low-volume subdural hematoma and ischemic stroke. Additional research and guidelines are necessary to manage such cases. A multidisciplinary approach comprising neurologists, neurosurgeons, and neuroradiologists is essential to customize treatment strategies for each patient’s specific needs. Furthermore, it is crucial to carefully consider approaches that address both coagulopathy management and optimal care of acute neurological conditions.

In conclusion, this case highlights the complex clinical challenges of managing a patient on anticoagulation therapy with a small subdural hematoma and ipsilateral ischemic stroke that was not initially visible on a CT scan. These challenges require a comprehensive and collaborative approach that encompasses the diagnostic, therapeutic, and preventive aspects.

## Data availability statement

The datasets presented in this article are not readily available because of ethical and privacy restrictions. Requests to access the datasets should be directed to the corresponding author.

## Ethics statement

Ethical review and approval was not required for the study on human participants in accordance with the local legislation and institutional requirements. Written informed consent to participate in this study was provided by the participants’ legal guardian/next of kin. Written informed consent was obtained from the participants’ legal guardian/next of kin for publication of this case report and any accompanying details and images.

## Author contributions

DS: Conceptualization, Data curation, Formal analysis, Funding acquisition, Investigation, Methodology, Project administration, Resources, Software, Supervision, Validation, Visualization, Writing – original draft, Writing – review & editing. AS: Conceptualization, Data curation, Formal analysis, Funding acquisition, Investigation, Methodology, Project administration, Resources, Software, Supervision, Validation, Visualization, Writing – original draft, Writing – review & editing. JB: Writing – original draft, Writing – review & editing. RR: Writing – original draft, Writing – review & editing. IV: Conceptualization, Data curation, Formal analysis, Funding acquisition, Investigation, Methodology, Project administration, Resources, Software, Supervision, Validation, Visualization, Writing – original draft, Writing – review & editing.
